# Profiling COVID-19 Genetic Research: A Data-Driven Study Utilizing Intelligent Bibliometrics

**DOI:** 10.3389/frma.2021.683212

**Published:** 2021-05-24

**Authors:** Mengjia Wu, Yi Zhang, Mark Grosser, Steven Tipper, Deon Venter, Hua Lin, Jie Lu

**Affiliations:** ^1^ Australian Artificial Intelligence Institute, Faculty of Engineering and Information Technology, University of Technology Sydney, Ultimo, NSW, Australia; ^2^ 23Strands, Pyrmont, NSW, Australia

**Keywords:** COVID-19, bibliometrics, network analytics, genetic research, knowledge discovery

## Abstract

The COVID-19 pandemic constitutes an ongoing worldwide threat to human society and has caused massive impacts on global public health, the economy and the political landscape. The key to gaining control of the disease lies in understanding the genetics of SARS-CoV-2 and the disease spectrum that follows infection. This study leverages traditional and intelligent bibliometric methods to conduct a multi-dimensional analysis on 5,632 COVID-19 genetic research papers, revealing that 1) the key players include research institutions from the United States, China, Britain and Canada; 2) research topics predominantly focus on virus infection mechanisms, virus testing, gene expression related to the immune reactions and patient clinical manifestation; 3) studies originated from the comparison of SARS-CoV-2 to previous human coronaviruses, following which research directions diverge into the analysis of virus molecular structure and genetics, the human immune response, vaccine development and gene expression related to immune responses; and 4) genes that are frequently highlighted include *ACE2*, *IL6*, *TMPRSS2*, and *TNF*. Emerging genes to the COVID-19 consist of *FURIN*, *CXCL10*, *OAS1*, *OAS2*, *OAS3*, and *ISG15*. This study demonstrates that our suite of novel bibliometric tools could help biomedical researchers follow this rapidly growing field and provide substantial evidence for policymakers’ decision-making on science policy and public health administration.

## Introduction

The COVID-19 pandemic has developed into an unprecedented global crisis that impacts daily lives and healthcare services provision of human beings. To stop its spread and efficiently control it, the biomedical research community has responded proactively on multiple fronts, including in the field of genetic research. By deciphering the genetic mechanisms underlying the body’s response to SARS-CoV-2 infection, we can arrive at a better understanding of COVID-19 pathogenesis, diagnosis, treatment, and, potentially, prevention, such as the optimization of vaccine development. In practical terms, multiple efforts on COVID-19 genetic research have resulted in a substantial amount of research publications (Chua et al., 2020; Shin et al., 2020; Wrobel et al., 2020; Pairo-Castineira et al., 2021). However, the downside of this productivity is that the quantity of COVID-19 literature proliferates and results in difficulties for researchers in comprehending this field’s changing knowledge landscape, particularly regarding the emerging information on various genes involved in COVID-19 response.

Bibliometrics is a subject that deciphers the patterns of scientific activities by quantitively tracking and measuring research activities. Traditional bibliometric approaches exploit statistical models to analyze bibliographic information such as author entities, keywords and citations and measure scientific activities. In the medical domain, such approaches have been successfully examined by multiple studies, such as profiling research landscapes (Thompson and Walker, 2015; Liao et al., 2018; Huang et al., 2019; Zhang L. et al., 2020) and discovering/inferring knowledge associations (Hristovski et al., 2005; Porter et al., 2020; Wu et al., 2021). Nowadays, artificial intelligence (AI) and data science techniques have empowered current bibliometrics with novel capabilities of excavating implicit knowledge and inferring potential knowledge associations from bibliometric data, which we named intelligent bibliometrics (Zhang et al., 2020c).

The intensive growth of COVID-19 publications has triggered considerable attention from the bibliometrics community. As part of those studies, Colavizza et al. (2021) profiled the trending research topics in the early-stage COVID-19 studies to indicate the initial foci of such studies, Chahrour et al. (2020) presented descriptive statistics of publication distribution and raised a call for more observational studies and therapeutic trials, Fry et al. (2020) revealed how COVID-19 has impacted and reshaped the worldwide scientific collaboration landscape, Zhang et al. (2021a) highlighted the disruption and resilience of research topics in coronavirus studies due to the outbreak of COVID-19. Different from other COVID-19 bibliometric studies, the study presented here leverages multiple approaches of intelligent bibliometrics and focuses specifically on the topic of COVID-19 genetic research. It utilizes multiple traditional and novel bibliometric analyses to profile the research landscape of this emerging field. This study particularly addresses the following questions:

1) Who are the key players in COVID-19 genetic research?
2) What research topics are prevalently addressed in COVID-19 genetic studies?
3) How have the foci of genetic studies changed during the COVID-19 crisis?
4) What specific genes are frequently highlighted and which ones are emerging as relatively newly-described entities that may be potentially important in COVID-19 genetic studies?

To answer these questions, we identified 5,632 COVID-19 genetic research articles within PubMed and applied a three-stage analysis, including 1) performing co-occurrence analysis to identify key players and core research topics in the field; 2) employing a topic tracking method named scientific evolutionary pathways (SEP) to trace the changing foci of these research topics over time during the COVID-19 pandemic period; 3) utilizing bio-entity network analytics to identify key genes and emerging genes in COVID-19 genetic research. The results also identify top research institutions in this field and their collaborating patterns, and some potential insights in terms of science, technology and innovation from the rapidly growing body of COVID-19 genetic research.

## Materials and Methods

**FIGURE 1:**
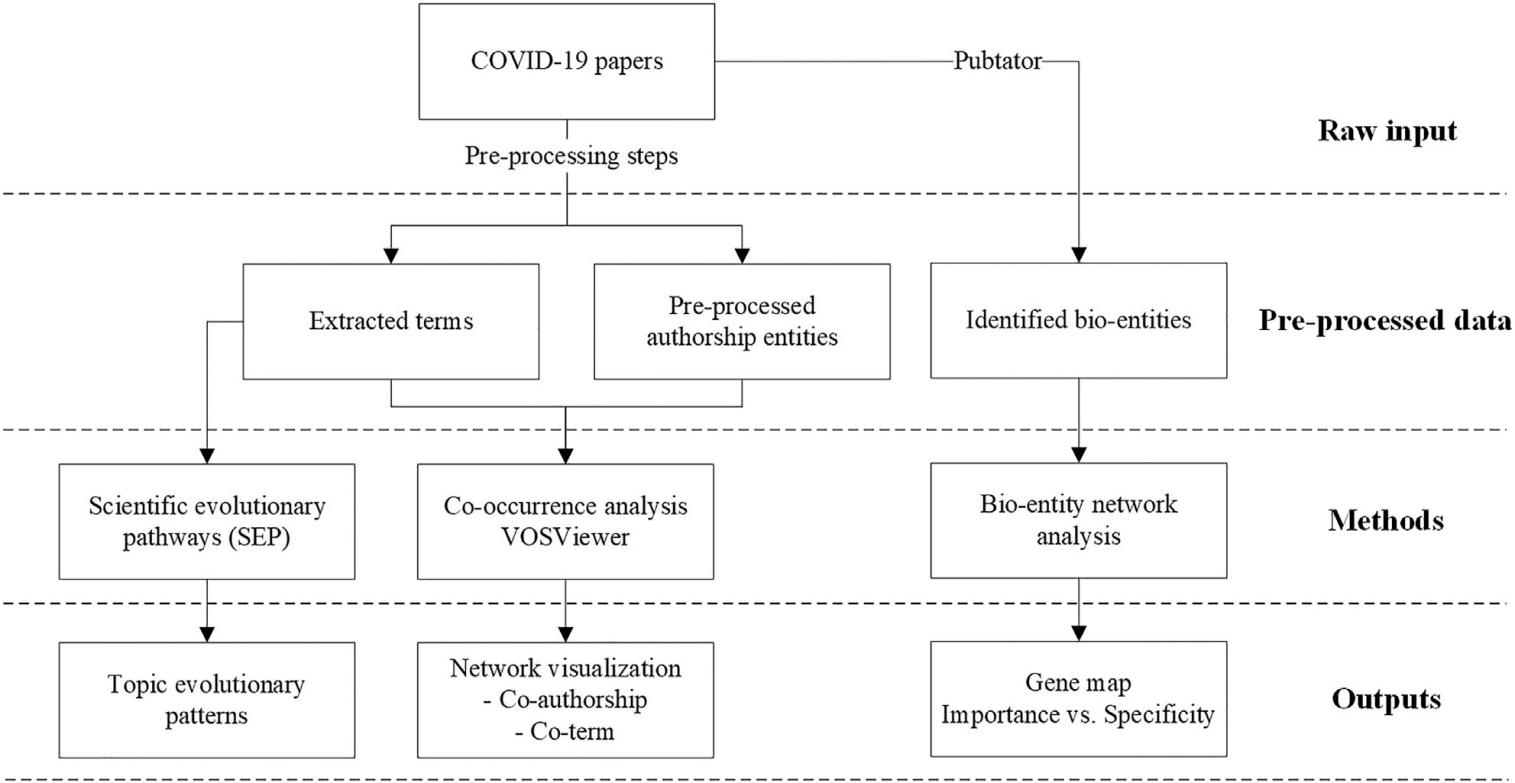
The research framework.

The research framework of this study is given in Figure 1.

### Data Collection

PubMed is a public biomedical literature database developed by the National Library of Medicine (NIH) and comprises over 32 million medical articles and online books.^1,^ ^2^ Falagas et al. (2008) recommend PubMed as the optimal bibliometric database for medical and life sciences, which exactly coheres with the foci of our study. Its advantage in biomedical information retrieval is providing specialized functions like Medical Subject Heading (MeSH) search and biomedical filters (including the species filter we used in this work) to return precise results. While considering Web of Science (WoS) is also a well-recognized data source in traditional bibliometric studies, we compared the search results of WoS and PubMed using the same search string (the filter and MeSH terms excluded) and noted that 93% of our collected data are indexed by WoS, indicating a wide coverage of PubMed data. Given that circumstances, in our study, we only exploited the PubMed database as our data source.

Using the search strategy below, we collected a dataset of the genetic research performed on COVID-19 and SARS-CoV-2 from PubMed:

(“"COVID-19/genetics”[MeSH Terms] OR ((“genes”[MeSH Terms] OR “genetics”[MeSH Terms] OR “gene”[All Fields] OR “genes”[All Fields] OR “genome”[All Fields] OR “genetics”[All Fields]) AND (“COVID-19”[All Fields] OR “SARS-Cov-2”[All Fields]))) AND (humans [Filter])

Search date: 08/03/2021

The search yielded 5,632 publications related to COVID-19 genetic research. We restricted the species to human since our primary goal is only to explore the important human genes act in COVID-19 infection.

### Key Player Identification and Research Landscape Profiling

Co-occurrence visualization, a type of science map, is a classical and effective way of profiling the research landscape in a certain field (Callon et al., 1983). The co-occurrence of authorship entities like authors, affiliations and countries reflects established collaborations at the individual, institutional or international levels. The co-occurrence of research content information like keywords or scientific terms in the same context indicates similar semantic meanings and their high probability of constituting a research topic. Visualization of such co-occurrence networks could straightforwardly highlight the key players or research communities in the field and reveal trending research topics in a specific field.

A co-occurrence network could be denoted as G=(V,E) where V represents the set of entities (authorship entities, terms, etc.) and E means the set of co-occurrences between entities. The co-occurrence network could also be represented as an adjacency matrix *A* with:

$$A_{i,j\left(i\neq j\right)}=\{\begin{array}{cc} CF\left(i,j\right) & if\;entity\;i\;and\;j\;co-occur\;in\;at\;least\;one\;document\\ 0 & otherwise \end{array}$$

where CF(i,j) means the frequency (i.e., number of publications) that entity *i* and *j* co-occur.

In the co-authorship network, considering the scale variation of different author entities, we will normalize $A_{i,j}$ by calculating the Jaccard Coefficient of *i* and *j* (Salton and McGill, 1986):

$$A_{i,j\left(i\neq j\right)}=\{\begin{array}{ll} \frac{CF\left(i,j\right)}{F\left(i\right)+F\left(j\right)-CF\left(i,j\right)}\; & if\;entity\;i\;and\;j\;co-occur\;in\;at\;least\;one\;document\\ 0 & otherwise \end{array}$$

where F(i) means the occurring frequency (i.e., number of publications) of entity i.

VOSViewer (Van Eck and Waltman, 2010) is utilized to visualize the co-occurrence networks of co-term and co-authorship. Note that all the parameters in VOSViewer are following its default settings (e.g., attraction and repulsion for layout, resolution and minimum cluster size for clustering). The local moving algorithm integrated in VOSViewer is simultaneously applied to identify communities in a network, which is reflected as the grouping and coloring strategy for its involved nodes.

### Scientific Evolutionary Pathways

Scientific Evolutionary Pathway (SEP), developed by Zhang et al. (2017), is a method of tracking the change of research topics retrieved from a collection of scholarly literature. The design of SEP adopts the assumption that scientific innovation results from the accumulative changes and recombination of existing knowledge (Fleming, 2001; Fleming and Sorenson, 2004). Algorithmically, SEP deems a document a collection of scientific terms and represents the document set as a document-term matrix. By dividing the literature dataset into sequential slices according to their publication gaps (year or month), SEP simulates scientific documents as streaming vectors of terms with a given vocabulary collected from the dataset and 1) extracts research topics via a K-means clustering algorithm, 2) captures semantic drifts of topics to identify topic evolution, and 3) represents topic evolutionary patterns by predecessor-descendant relationships between topics. The stepwise algorithm is given as follows:

Concept definition: A topic is denoted as *T* and defined as a collection of articles represented by term vectors. Every topic has two attributes: 1) the centroid *c*, which is represented by the mean vector of all the articles that belong to the topic; and 2) the radius *r*, which is represented by the largest Euclidean distance of the centroid to every article in *T*.

Step 1: Initialize the document-term matrix and divide the documents into time slices according to their publishing dates.

Step 2: Group articles in the first time slice (Slice 0) to an initial topic $T_{i}$ and label it with the top-frequency term, then calculate its radius *r* and centroid *c*.

Step 3: For every article a in the next time slice (Slice 1), calculate the article’s Euclidean distance E(a,c) between a and the topic’s centroid *c*. If (E(a,c)−r)/r>σ (σ is a given threshold which is set as 0.1 by default), it will be recognized as a drifted article to $T_{i}$, otherwise the article will be classified as belonging to $T_{i}$.

Step 4: Once all articles in Slice 0 have been processed, update the centroid and radius of $T_{i}$ with these newly added articles. For the drifted articles, use a K-means clustering algorithm to form a new topic set $\left\{T_{n}\right\}$. All topics in $\left\{T_{n}\right\}$ will be deemed as the descendent topics of $T_{i}$.

Step 5: For the forthcoming time slices, iterate Steps 3–4 until the last slice of the entire dataset. However, starting from Slice 2, we will measure an article’s similarity with not only $T_{i}$ but all the existing topics (e.g., $\left\{T_{n}\right\}$), and assign the article to the most similar topic based on Salton’s cosine similarity.

The outcome of SEP analysis is a set of topics with a time stamp indicating when it was born and with directed links representing their predecessor-descendent relationships.

### Bio-Entity Network Analysis

Bio-entity network analysis is a method for literature-based discovery proposed by Wu et al. (2021), which is used to discover and infer genetic knowledge for a specific disease. The method extracts bio-entity concepts from literature and constructs a heterogeneous bio-entity co-occurrence network. Further, it presents the extracted bio-entities comprehensively based on their topological importance and specificities to the target disease. In our case, we select disease, chemical, gene and genetic variant as four representative bio-entities, and the co-occurrence network is denoted as:

$$G=\left(V^{d,c,g,v},E^{pairwise\left(d,c,g,v\right)}\right)$$

where d,c,g,v respectively represent the nodes of diseases, chemicals, genes and genetic variants.

$$w_{V_{i}V_{j}\left(i\neq j\right)}=\{\begin{array}{ll} CF\left(V_{i},V_{j}\right) & if\;V_{i}\;and\;V_{j}\;co-occur\;in\;at\;least\;one\;document\\ 0 & otherwise \end{array}$$

where $w_{V_{i}V_{j}\left(i\neq j\right)}$ represent the weight of edge linking $V_{i}$ and $V_{j}$.

Three node centralities are employed to measure the importance of individual nodes in the whole network. Referring to the discussion given by Zhang et al. (2021), among the three centralities, *degree centrality (DC)* measures a node’s ability to aggregate information, representing its local influence within a network; *closeness centrality (CC)* measures a node’s ability to disseminate information, as well as its global influence on all other nodes within a network; *betweenness centrality (BC)* represents a node’s ability to act as a bridge between diverse information content, which calculates the proportion of shortest paths going through the node. The three centralities can be calculated as follows:

$$DC\left(V_{i}\right)=\frac{\sum_{k\in \left\{d,c,g,v\right\}}^{k}\sum_{j=1}^{\left|V^{k}\right|}\left(1\;if\;w_{V_{i}V_{j}^{k}\left(i\neq j\right)}\;else\;0\right)}{\left|V^{d,c,g,v}\right|-1}$$

where $V_{i}$ is the target node, $V_{j}^{k}$ is the *j*th node in category k∈{d,c,g,v}, $\left|V^{k}\right|$ is the number of nodes in the node set $V^{k}$, $\left|V^{d,c,g,v}\right|$ is the number of nodes in the total node set.

$$CC\left(V_{i}\right)=\frac{\left|V^{d,c,g,v}\right|-1}{\sum_{k\in \left\{d,c,g,v\right\}}^{k}\sum_{j=1}^{\left|V^{k}\right|}d_{V_{i}V_{j}^{k}}}$$

where $d_{V_{i}V_{j}^{k}}$ denotes the shortest topological distance between $V_{i}$ and $V_{j}^{k}$.

$$BC\left(V_{i}\right)=\frac{2\sum_{x,\;y\in \left\{d,c,g,v\right\}}^{x,y}\sum_{a=1}^{\left|V^{x}\right|}\sum_{b=1}^{\left|V^{y}\right|}\frac{\sigma {\left(V_{a}^{x}V_{b}^{y}\right)}_{V_{i}}}{\sigma \left(V_{a}^{x}V_{b}^{y}\right)}}{\left(\left|V^{d,c,g,v}\right|-1\right)\left(\left|V^{d,c,g,v}\right|-2\right)}\left(V_{i}\neq V_{a}^{x}\neq V_{b}^{y}\right)$$

where $\sigma \left(V_{a}^{x}V_{b}^{y}\right)$ denotes the number of shortest paths between $V_{a}^{x}$ and $V_{b}^{y}$, $\sigma {\left(V_{a}^{x}V_{b}^{y}\right)}_{V_{i}}$ is the number of shortest paths between $V_{a}^{x}$ and $V_{b}^{y}$ that pass through $V_{i}$.

Apart from the centralities that reflect a node’s importance, the method also utilizes another indicator named intersection ratio to measure whether a gene is specific to the target disease. The intersection ratio is calculated as follows:

$$IR\left(V_{i}\right)=\frac{w_{V_{i}V_{d}^{t}}}{\sum_{j=1}^{V^{d}}w_{V_{i}V_{j}^{d}}}$$

where $V_{d}^{t}$ is the target disease, $V^{d}$ is the set of disease nodes.

Three centralities together constitute the topological importance of a node in a network. To comprehensively rank this importance, we exploit an entropy-based algorithm (Grupp, 1990) to combine those centralities in a data-driven way. Briefly, we initially normalize the values of each centrality using the min-max normalization approach and then calculate the entropy of each normalized centrality and get its weight, respectively. The combined centrality of every individual node can eventually be finalized with those data-driven weights. Using *DC*, the value set of the degree centrality, as an example, we calculate the weight of *DC* as follows:

Step 1: Normalize each centrality value set:

$$nDC\left(V_{i}\right)=\frac{DC\left(V_{i}\right)-\text{min}\left(DC\right)}{\text{max}\left(DC\right)-\text{min}\left(DC\right)}$$

where *DC* is the value set of the degree centrality, max(DC) and min(DC) is respectively the maximum and minimum value in this set.

Step 2: Calculate the entropy of each centrality:

$$H_{DC}=-\frac{1}{\ln\left|\text{V}\right|\;}\sum_{i}^{\left|\text{V}\right|\;}nDC\left(V_{i}\right)\text{ln}\left(nDC\left(V_{i}\right)\right)\;$$

where V is the collection of all nodes in the given network.

Step 3: Calculate the weight of each centrality according to the entropy, when we get the entropy of all three centralities:

$$w_{DC}\;=\frac{1-H_{DC}}{3-\left(H_{DC}+H_{BC}+H_{CC}\right)}\;\left(0\leq w_{DC}\leq 1\right)$$

Step 4: Finalize the centrality combination value for every individual node:

$$Centralities\;combination\left(V_{i}\right)=w_{DC}nDC\left(V_{i}\right)+w_{CC}nCC\left(V_{i}\right)+w_{BC}nBC\left(V_{i}\right)$$

## Results

To conduct the analyses above in a systematic manner, we utilized a one-stop platform that integrates multiple bibliometric methods developed in our pilot studies (Zhang et al., 2020d; Wu et al., 2020). This platform contains six modules, including PubMed/PMC data import filter, bibliographic information statistics, co-occurrence analysis, research collaboration prediction, semantic similarity-based document search and SEP analysis. By feeding the raw data from PubMed into our bibliometric platform, we could efficiently generate results and then present these graphically with the aid of multiple visualizing tools, such as Gephi and VOSViewer.

### Key Players and Topics Identification

**FIGURE 2:**
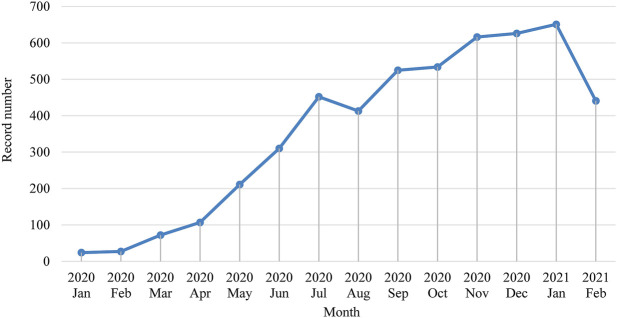
Monthly trend of the number of publications.

Apart from 623 publications that either miss publication month information or published after February 2021, we presented the monthly change of the numbers of publications in Figure 2. We could see a clear overall blooming trend of publications since January 2020. The downward trend starting in August 2020 may indicate that the stage shifts of COVID pandemic status has an influence on related academic research.

We implemented a set of pre-processing and disambiguation approaches to clean affiliation names and terms retrieved from combined titles and abstracts. Ranking by the total number of publications, we present the top 20 countries, research institutions and journal names in Table 1. At the country level, the United States and China are still leading this domain, followed by the United Kingdom, Italy, Germany, etc. From the institutional perspective, the top 20 most prolific institutions consist of eight institutions from the United States, six from China, four from the United Kingdom, and one each from Canada and Italy (the other major pandemic center in early 2020, besides China). Interestingly, compared to China’s leadership in pioneering general COVID-19 research at the early stage of the COVID-19 pandemic (Fry et al., 2020), prestigious universities from the United States and United Kingdom have become leaders in this domain, particularly in COVID-19 genetic research. The journal distribution of COVID-19 genetic research reflects that virology journals published the largest number of papers, and prestigious journals such as *Nature*, *Science*, *Cell* and *PNAS* also show an inclined favor for such studies.

**TABLE 1:** Top 20 prolific countries, research institutions and journals in this emerging field.

Ranking	Country	Research Institution	Journal
1	United States (1811)	University of California (183)—United States	Journal of Medical Virology (150)
2	China (1,030)	University of Texas (104)—United States	PLoS One (130)
3	United Kingdom (560)	University of Hong Kong (93)—China	Scientific Reports (100)
4	Italy (534)	University of Oxford (92)—United Kingdom	Nature (88)
5	Germany (359)	Wuhan University (76)—China	Viruses (82)
6	India (320)	University of Washington (76)—United States	Nature Communication (70)
7	France (299)	University of Pennsylvania (71)—United States	Frontiers in Immunology (70)
8	Canada (274)	Stanford University (63)—United States	International Journal of Infectious Diseases (68)
9	Spain (214)	University College London (59)—United Kingdom	Science (67)
10	Australia (196)	University of Cambridge (58)—United Kingdom	Emerging Microbes & Infections (64)
11	Brazil (173)	Massachusetts General Hospital (58)—United States	Journal of Clinical Virology (62)
12	Japan (150)	University of Chinese Academy of Sciences (55)—China	Medical Hypotheses (62)
13	Switzerland (141)	Tongji Hospital (54)—China	International Journal of Molecular Sciences (57)
14	Iran (140)	Columbia University (52)—United States	Cell (50)
15	South Korea (115)	University of Toronto (52)—Canada	Journal of Clinical Microbiology (46)
16	Belgium (105)	University of Edinburgh (51)—United Kingdom	Infection, Genetics and evolution (45)
17	Turkey (98)	University of Milan (51)—Italy	Signal Transduction and Targeted Therapy (44)
18	Sweden (88)	Brigham and Women’s Hospital (51)—United States	Proceedings of the National Academy of Sciences of the United States of America (42)
19	Saudi Arabia (67)	Peking Union Medical College (50)—China	BMC Infectious Diseases (41)
20	Austria (67)	Fudan University (48)—China	Eurosurveillance (39)

**FIGURE 3:**
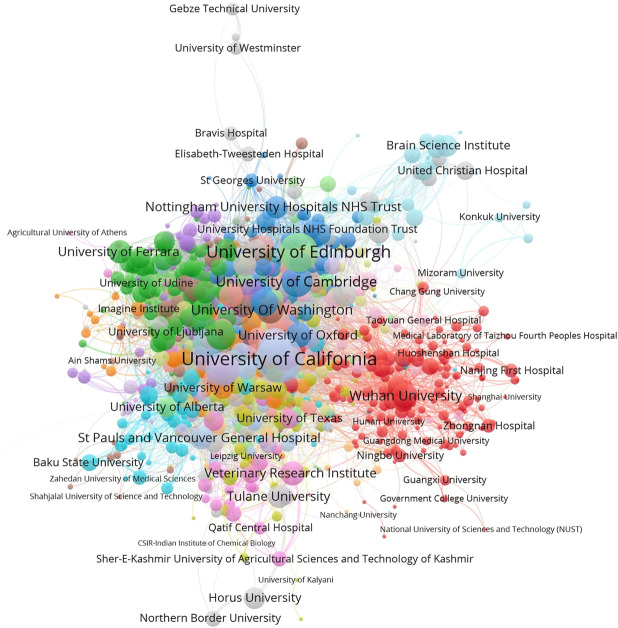
The co-authorship network of research institutions (normalized by Jaccard Coefficient).^2^

**FIGURE 4:**
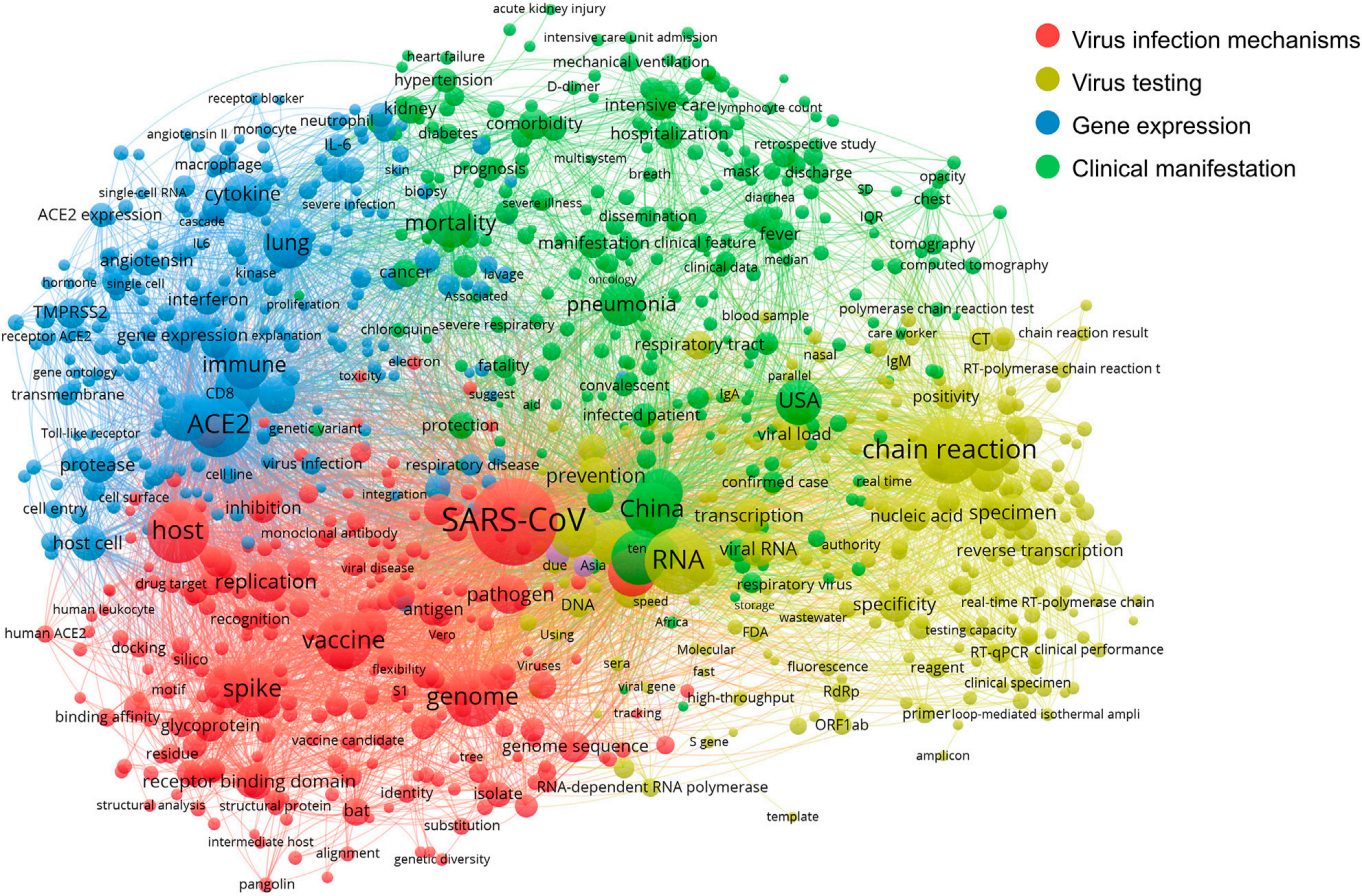
The co-occurrence network of scientific terms.

Then we conducted co-occurrence analysis to cleaned affiliations and terms and generated a co-authorship network and a co-term network. With the aid of VOSviewer, the visualizations of the co-authorship network and word co-occurrence network are shown in Figures 3, 4.

As shown in Figure 3, we can observe global collaborating patterns on COVID-19 genetic research. When the size of each node is measured by the sum of Jaccard Coefficients that the represented institution has with all its collaborators, it then reflects its representing institution’s collaborative strength in this field. Located at the center of this network, United States universities, such as the University of California systems and the University of Washington, have established broad and close international collaborations with worldwide institutions during the crisis. Research institutions from Europe and the United Kingdom share intensive partnerships with United States institutions. Comparably, Chinese institutions show strong domestic collaborations but have relatively sparse links with their international counterparts. Political issues might be a hidden reason behind this phenomenon, but the national border restrictions may play a crucial role in limiting collaborations within relatively small but well-established groups (Fry et al., 2020; Cai et al., 2021).

In Figure 4, the co-term network is partitioned into four communities with different colors: Virus infection mechanisms (red), virus testing (yellow), gene expression related mainly to the immune response to COVID-19 (blue) and COVID-19 clinical manifestation (green).

The network shows the relatedness and relationships within and between these four main areas of scientific efforts. The red community mostly covers molecular concepts related to the mechanism of virus infection, like the virus *spike protein S1*, *antigen*, *pathogen*, *host* and *vaccine*. The yellow community groups terms related to testing techniques such as *RNA*, *nucleic acid*, *RT-qPCR* and *chain reaction*. The blue community indicates gene expression, explicitly demonstrating genes and proteins involved in this broad area of research, including immune system cell types (macrophage; neutrophil), cell components (cell surface), cell surface markers (CD8), and immune response genes (IL6; interferon). Lastly, terms in the green community mostly describe the clinical phenotypes and manifestation of COVID-19 and epidemiological information, including *China*, the *United States* and *mortality*.

### Topic Evolution

**FIGURE 5:**
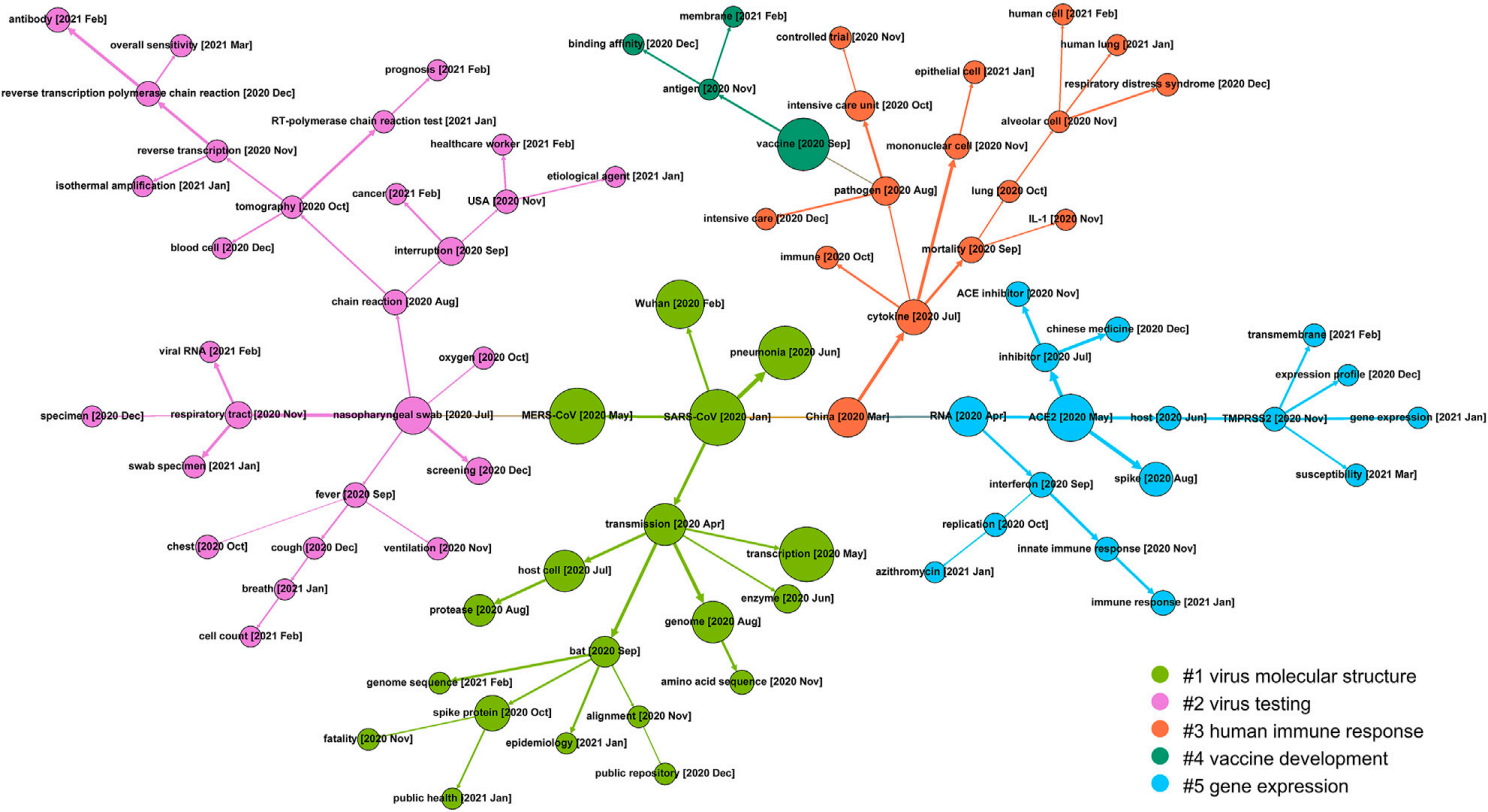
The SEP on COVID-19 genetic research between January 2020 and April 2021.

The SEP approach was applied to the 5,166 publications before April 2021. With the aid of Gephi (Bastian et al., 2009), we visualized the SEP on COVID-19 genetic research between January 2020 and April 2021 and present it in Figure 5. In this figure, 85 nodes are generated and linked by directed edges, representing their predecessor-descendant relationships, i.e., evolutionary patterns. Every topic name is followed by a bracketed time label, indicating when the topic was born. The descriptive statistics of the SEP is given in Table 2. We further applied an approach of community detection integrated in Gephi to group these topics into communities (with colors).

**TABLE 2:** The basic statistics of 85 topics.

Node number	Maximum publication number^a^	Minimum publication number^a^	Standard deviation
85	321	3	84.374
	MERS-CoV [2020 January]—321	Etiological agent [2021 January]—3	
	SARS-CoV [2020 January]—320	Human lung [2021 January]—3	
	Transcription [2020 May]—309	Cell count [2021 February]—3	
	Pneumonia [2020 June]—208	Healthcare worker [2021 February]—3	

a We list the top four topics that contain the largest/smallest numbers of publications in the table.

As given in Figure 5, during the 15 months, topics on COVID-19 genetic research have evolved into five communities, that is, #1 virus molecular structure, #2 virus testing, #3 human immune response, #4 vaccine development, and #5 gene expression.

Tracing back to the early stage of COVID-19 genetic research, most of the studies focus on the genetic similarity of COVID-19 with previous prevalent coronaviruses like severe acute respiratory syndrome coronavirus (*SARS-CoV*) and Middle East respiratory syndrome coronavirus (*MERS-CoV*). With this as a starting point, community #1 (the green branch) maps research on the molecular structure of viruses, including its probable source of origin (*bat*), *genome sequence*, how the virus invades the *host cell* and how the *spike protein* acts during the infection process, etc. Community #2 (the purple branch) details another research direction of COVID-19 testing and screening and covers a wide variety of topics like clinical manifestations (*fever*, *cough*, *cell count*), testing method (*viral RNA* testing) and techniques (*reverse transcription-polymerase chain reaction test*, which is regarded as the “gold standard” of virus RNA testing), test *sensitivity*, etc. This community reflects that genetic research on virus testing gathers substantial attention in the mid-term research of COVID-19. Community #3 (the red branch) on the right side concentrates on the body reaction of humans to COVID-19 infection. This community consists of concepts detailing the clinical manifestations and immune reactions in human cells. For example, the *cytokine* storm, which is postulated to be one of the major reasons for severe disease progression, is the result of an overreaction of the human immune system. Community #4 *vaccine* development is derived from the branch of immunological research and becomes a relatively outlying research focus. Along with this timeline, the specific process within human cells is also frequently studied as presented in community #5 gene expression, where *ACE2* and *TMPRSS2*, two of the key genes involved in the viral infection process, are highlighted.

### Bio-Entity Network Analysis

#### Entity Extraction and Pre-processing

We utilized Pubtator (Wei et al., 2019), a deep learning-based entity extraction tool developed by the National Library of Science (NLM), to extract biomedical concepts from titles and abstracts of the collected research papers. The raw output from Pubtator is lists of biomedical concepts with exclusive identifiers. We then employed multiple biomedical dictionaries, including Medical Subject Headings,^3^ NCBI gene dictionary of *Homo sapiens*,^4^ as well as a single nucleotide polymorphism (SNP) dictionary from the dbSNP database,^5^ to map those concepts to unified bio-entities.

The extraction process resulted in 48,201 raw biomedical concepts, including diseases, chemicals, genes and genetic variants. We then mapped every concept to its related dictionary and applied two cleaning steps to remove noise (Step 1) and consolidate synonyms (Step 2). 2,573 unique bio-entities were retrieved after the cleaning steps, with the stepwise results presented in Table 3. The 2,573 bio-entities were then used to construct a heterogeneous co-occurrence network.

**TABLE 3:** Stepwise results of the pre-processing procedure.

	Raw	Step 1	Cleaned	Step 2	Nodes
Disease	31,974	Removed noisy concepts like “cardioembolic”, “JAGS”, “nonvitamin”, etc. that could not be mapped to MeSH	31,963	MeSH	801
Chemical	4,494		3,724		678
Gene	11,211	Excluded genes that do not belong to *Homo-sapiens*	8,781	NCBI Gene	968
Gene variant					
DNA mutation	69	Removed variants with unclear loci (i.e., could not be mapped to an SNP ID)	17	dbSNP	126
Protein mutation	349		91		
SNP	104	—	104		
Total	48,201	—	44,680	—	2,573

The co-occurrence network of the 2,573 entities contains 31,848 edges. The counts of different types of edges are given in Table 4.

**TABLE 4:** Counts of the different types of edges.

	Disease	Chemical	Gene	Genetic variant
Disease	8,231	4,872	6,966	499
Chemical	4,872	2,121	2,268	37
Gene	6,966	2,268	5,692	385
Genetic Variant	499	37	385	777

#### Profiling Bio-Entities

**FIGURE 6:**
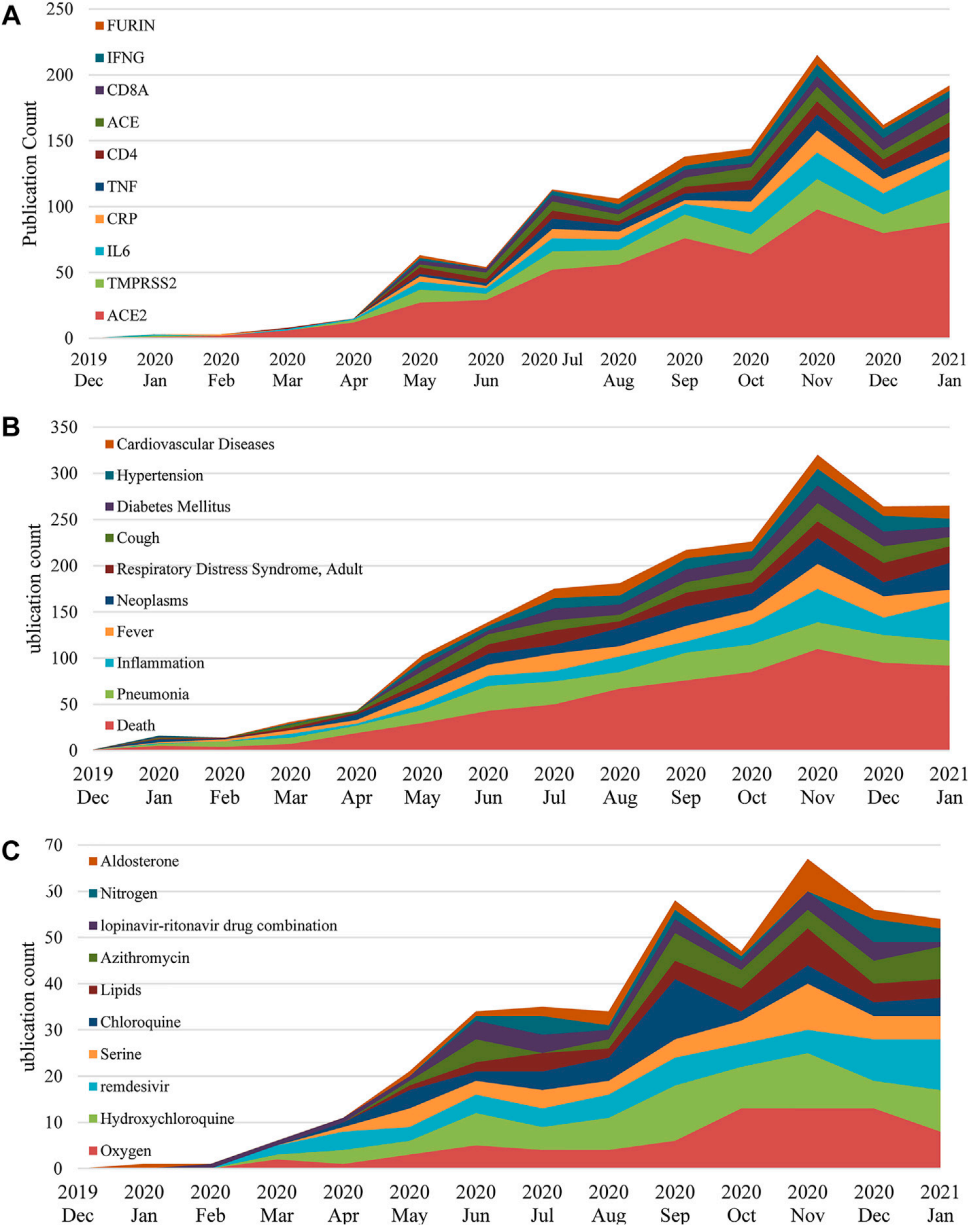
Top 10 genes **(A)**, co-morbidities **(B)** and chemicals **(C)** in COVID-19 genetic research between December 2019 and January 2021.

Based on the frequencies of those identified bio-entities, we list the top 10 highly frequent bio-entities in Table 5. The monthly changes of those entities regarding their frequency are provided in Figure 6. Genetic variation is not given due to the relatively small amount of data in this category. We only traced the data to January 2021 since the latter collection is incomplete due to the publishing lags.

**TABLE 5:** The top 10 entities ranked by the raw frequency.

Ranking	Disease	Chemical	Gene	Genetic variant
1	Death	Oxygen	*ACE2*	rs2285666
2	Pneumonia	Hydroxychloroquine	*TMPRSS2*	rs12329760
3	Inflammation	Remdesivir	*IL6*	rs4646116
4	Fever	Serine	*CRP*	rs11385942
5	Neoplasms	Chloroquine	*TNF*	rs12252
6	Respiratory distress syndrome, adult	Lipids	*CD4*	rs1244687367
7	Cough	Azithromycin	*ACE*	rs143936283
8	Diabetes mellitus	Lopinavir-ritonavir drug combination	*CD8A*	rs73635825
9	Hypertension	Nitrogen	*IFNG*	rs8176746
10	Zoonoses	Aldosterone	*FURIN*	rs8176719

In general, the frequencies of the top 10 entities in each category keep increasing during the pandemic. Predictably, ACE2 is the top mentioned gene with a noticeable frequency gap with the following genes in Figure 6A. This is mainly because ACE2 is the primary functional receptor for the SARS-CoV-2 virus in human cells (Zheng Y.-Y. et al., 2020). Next, Figure 6B profiles co-morbidities discussed in COVID-19 genetic studies, besides those symptoms and COVID-19 manifestations, such as cough, respiratory distress syndrome and inflammation, key co-morbidities in COVID-19 genetic studies include neoplasms, diabetes and hypertension. Noteworthy, Figure 6C presents multiple prevalent drug treatments that were utilized and trialed for COVID-19, including hydroxychloroquine, azithromycin, remdesivir and the lopinavir-ritonavir drug combination, indicating that the pharmacogenomics of those drugs is also a major interest of this domain.

#### Emerging Gene Discovery

**FIGURE 7:**
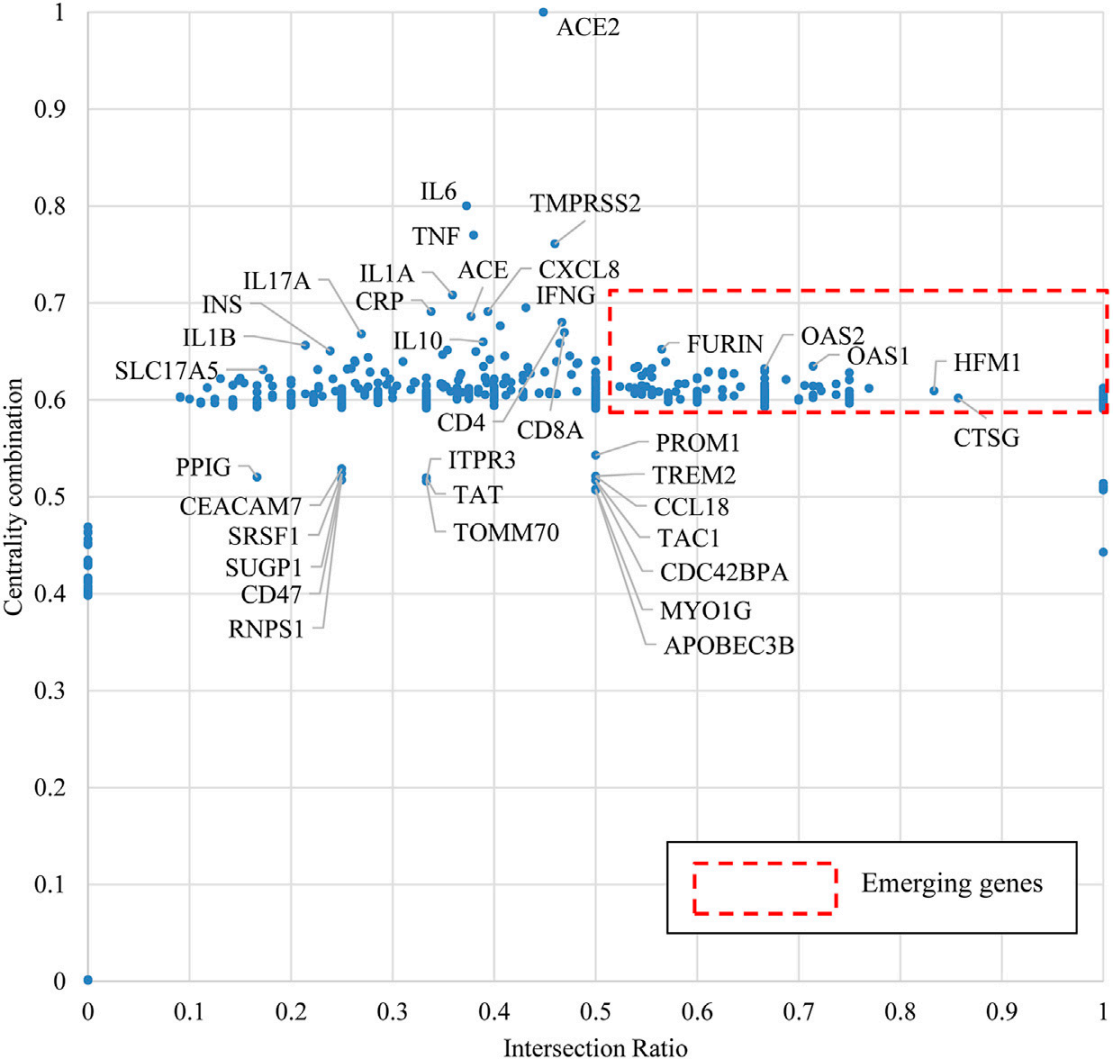
Emerging gene discovery for COVID-19 genetic research.

We applied the approach of bio-entity network analytics and attributed each node of genes with two indicators: centrality combination and intersection ratio. We then normalized the two indicators as *X*-axis Intersection Ratio and *Y*-axis Centrality Combination and located all genes in a coordinate system in Figure 7. According to our design, a high value of centrality combination may indicate the importance/impact of a given gene to relatively broad domains of the target disease, while a high value of intersection ratio may represent the specialty of a given gene to the target disease.

**FIGURE 8:**
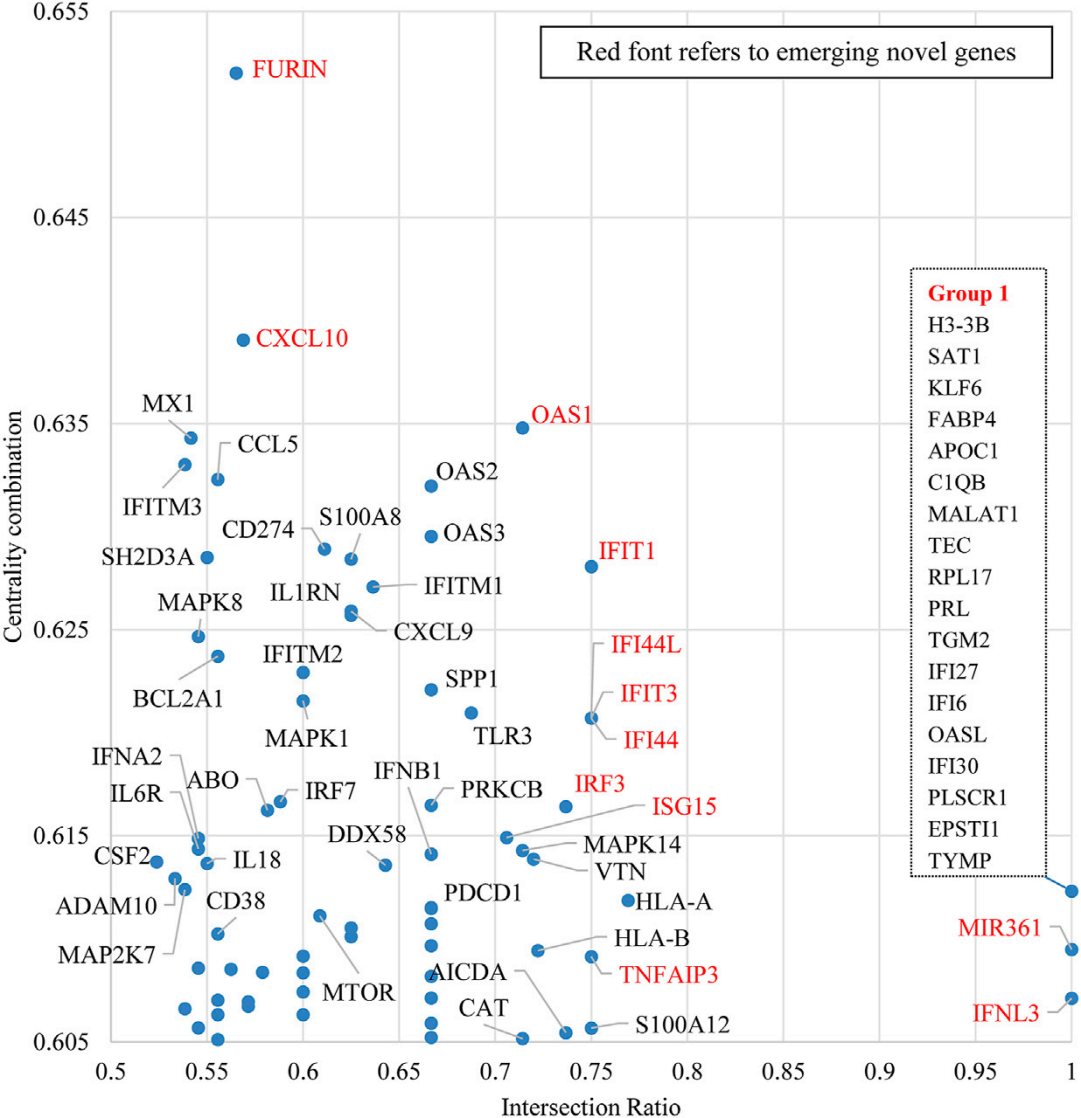
Emerging gene discovery for COVID-19 genetic research–detailed partial view. Note: This figure is a zoom-in map for the red broken-line box in Figure 7.

When COVID-19 is the target disease of our study, the centrality combination indicates the strength of a gene’s contribution to broad COVID-19 related concepts, while the intersection ratio reveals the strength of a gene’s specific relationship to COVID-19. From the perspective of centrality combination, we could identify *ACE2*, *IL6*, *TMPRSS2*, and *TNF* as the set of frequently highlighted genes. ACE2 is the major functional receptor for the SARS-CoV-2 virus (Zheng Y.-Y. et al., 2020). TMPRSS2 is an enzyme that primes the spike S protein of the SARS-CoV-2 virus to promote virus entry (Hoffmann et al., 2020b), IL6 and TNF are pro-inflammatory cytokines that are found generally elevated in severe COVID-19 patients (Cao, 2020). From the perspective of intersection ratio, we present Figure 8 to zoom into those emerging genes.

Figure 8 zooms in to some recently mentioned genes in the literature, which own both high intersection ratio and centrality combination in the first quadrant of Figure 7. The potential role of these “emerging” genes and their products in COVID-19 is discussed below:

*FURIN*: FURIN is an essential cleavage enzyme for the spike protein of SARS-CoV-2 in the virus infection process. From the biochemical perspective, Klimstra et al. (2020) identified the association between a putative furin cleavage signal generated by a novel insertion of the SARS-CoV-2 spike S glycoprotein and the expanded host range. Wrobel et al. (2020) discovered that the cleavage at the furin-cleavage site decreases the overall stability of SARS-CoV-2 S and facilitates the adoption of the open conformation required for the viral S (spike) protein to bind to the ACE2 receptor. From the treatment perspective, Hoffmann et al. (2020a) highlighted that obtaining a S1/S2 multibasic cleavage site was essential for COVID-19 infection and indicated furin as a potential target for therapeutic intervention. A similar finding was also presented by Sallenave and Guillot (2020), whose study identified a furin-like cleavage site in SARS-CoV-2 to facilitate the S protein priming, they also claimed that furin inhibitors can be targeted as potential drug therapies for SARS-CoV-2.

*CXCL10*: *CXCL10* is a frequently studied gene in multiple COVID-19 genetic studies (Bermejo-Martin et al., 2020; Chua et al., 2020; Hou et al., 2020; Parkinson et al., 2020; Xiong et al., 2020; Han et al., 2021; Tan et al., 2021). Among those studies, a paper published on *Nature Biotechnology* identified that critical COVID-19 cases had shown stronger interactions between epithelial and immune cells which includes inflammatory macrophages expressing CXCL10 (Chua et al., 2020). Bermejo-Martin et al. (2020) reported that viral RNA load in plasma correlates with higher chemokines levels, including CXCL10 and CCL2. Xiong et al. (2020) also indicated the association between COVID-19 pathogenesis and excessive cytokine release, including CXCL10/IP-10.

*OAS1, OAS2, OAS3, IFIT1, IFIT3, IFI44, IFI44L and IFITM1*: Current COVID-19 genetic studies incline to analyze those genes together. In a paper published in *Nature*, Pairo-Castineira et al. (2021) identified a significant genetic variant rs10735079 associated with critical illness of COVID-19 in the gene cluster encodes *OAS1*, *OAS2*, and *OAS3*. Interestingly, recent work on archaic human (Neandertal) DNA has identified an additional haplotype in the region of Chromosome 12 containing *OAS1*, *OAS2*, and *OAS3* that protects against severe COVID-19 (Zeberg and Paabo, 2020). Klaassen et al. (2020) identified six genetic variants in innate immunity-related genes, including *OAS1* (p.Arg130His), which might have predictive value for COVID-19 infection. Besides, *IRF9, IFIT1, IFITM1, MX1, OAS2, OAS3, IFI44* and *IFI44L* were found to be upregulated in the COVID-19 infected normal human bronchial epithelial cells (Vishnubalaji et al., 2020). Similarly, Prasad et al. (2020) also found that some interferon-stimulated genes can be considered as potential candidates for drug targets in COVID-19 treatment. Those genes include *IFIT1, IFITM1, IRF7, ISG, MX1*, and *OAS2*. Shi et al. (2021) showed that COVID-19 infections are generally restricted by IFITM1, IFITM2 and IFITM3 using gain- and loss-of-function approaches.

*ISG15*: The findings of ISG15 are mostly related to the papain-like proteases (PLpro) encoded by the SARS-CoV-2 coronavirus. A paper published in *Nature* revealed a unique preference of SARS-CoV-2 coronavirus of cleaving ubiquitin-like interferon-stimulated gene 15 protein (ISG15), which is different from SARS-CoV (Shin et al., 2020). This study also indicated that SARS-CoV-2 papain-like protease contributes to the cleavage of ISG15 from interferon responsive factor 3 (IRF3) and attenuates type I interferon responses. Klemm et al. (2020) specified that the structure of the SARS-CoV-2 PLpro reveals that S1 ubiquitin-binding site is responsible for high ISG15 activity, while the S2 binding site provides Lys48 chain specificity and cleavage efficiency. Freitas et al. (2020) evaluated the biochemical activity of SARS-CoV-2 PLpro and ISG15 with its counterparts in MERS-CoV and SARS-CoV. They indicated that naphthalene based PLpro inhibitors are shown to be effective at halting SARS-CoV-2 PLpro activity as well as SARS-CoV-2 replication.

*IRF3*: Zheng et al. (2020b) found that the interaction of COVID-19 M-protein with RIG-1, MAVS, and TBK1 inhibits the formation of multiprotein complexes of those proteins encoded by these genes and subsequently prevents the activation of IRF3. Moustaqil et al. (2020) Identified COVID-19 NSP3 and NSP5 protease’s new functions of specifically and selectively cleaving IRF-3, NLRP12 and TAB1. Lévy et al. (2021) reported a case of IFN-α2a therapy in two patients with inborn errors of TLR3 and IRF3 infected with COVID-19. Yin et al. (2020) found that IRF3, IRF5 and NF-κB/p65 are the key transcription factors regulating the IFN response during SARS-CoV-2 infection.

*TNFAIP3*: Protein and protein interaction analysis from Islam et al. (2020) indicated that *TNFAIP3* is one of the kye hub genes that have good binding affinities with repurposed COVID-19 drug candidates which includes dabrafenib, radicicol and AT-7519. Li et al. (2021) observed the bimodal gene expression of *TNFAIP3* in various immune cells from severely infected COVID-19 patients.

The overlapping genes in Group 1 are investigated in a single paper (Shaath et al., 2020). They identified neutrophils (*IFITM2, IFITM1, H3-H3B, SAT1* and *S100A8*) and macrophage cluster-1 (*CCL8, CCL3, CCL2, KLF6* and *SPP1*) as the main immune cell subsets associated with severe COVID-19 cases. They also found that some upstream regulators (IFNG, PRL, TLR7, PRL, TGM2, TLR9, IL1B, TNF, NFKB, IL1A, STAT3, CCL5) were enriched in bronchoalveolar lavage cells in severe COVID-19 cases compared to the mild cases. Besides, a number of genes found in both mild and severe COVID-19 cases (*IFI27, IFITM3, IFI6, IFIT3, MX1, IFIT1, OASL, IFI30, OAS1*) and genes only in severe cases (*S100A8, IFI44, IFI44L, CXCL8, CCR1, PLSCR1, EPSTI1, FPR1, OAS2, OAS3, IL1RN, TYMP, BCL2A1*) are reported as well.

*MIR361*: miRNAs are important regulators of viral pathogenesis, particularly among RNA viruses. Pierce et al. (2020) verified the biological plausibility of the predicted miRNA-target RNA interactions, in which miRNA361 binds to the SARS-CoV-2 IFN-α 3′-UTR. Li et al. (2020) showed that hsa-miR-361-3P is one of the top upregulated or downregulated genes in COVID-19 patients compared to the healthy controls.

*IFNL3*: Stevenson et al. (2021) claimed IFNL3 as one of the predictive markers for severe symptoms of COVID-19 based on analysis of serum chemokines and cytokines from COVID-19 patents, while another pharmacogenomic study did not find the potential of IFNL3 in modifying treatments (Sugiyama et al., 2020). Instead, it identifies CYP2D6 and CYP2C19 as the two likely best targets for treatment modification, especially by ondansetron, oxycodone, and clopidogrel.

## Discussion and Conclusion

The COVID-19 pandemic remains a worldwide threat to human health, the global economy and the political landscape. Controlling the pandemic and strengthening disease prevention and treatment could be the top priority of the entire globe. Despite hopes from vaccine rollouts, there is still substantial knowledge about this virus waiting for discovery and understandings. Whereas a large number of studies on COVID-19 genetic research has been done since the beginning of the COVID-19 pandemic, keeping up with this rapid change is challenging scientific researchers and policymakers. This paper comprehensively analyzed research papers on COVID-19 genetic research published during this pandemic period. We incorporated co-occurrence analysis, scientific evolutionary pathways, and bio-entity network analytics to conduct multi-dimensional tasks of knowledge discovery, particularly with the following four research questions:

Q1: Who are the key players in COVID-19 genetic research?

Universities from the United States are leading this domain, and research institutions from China, the United Kingdom, and Canada closely follow. Investigations on their collaboration patterns reveal among those leading countries, China tends to have strong domestic collaborations while other countries incline to establish international collaborations with each other.

Q2: What are the key research topics prevalently addressed in COVID-19 genetic research?

Figure 4 reveals four major research topics in this field, that is, virus infection mechanisms, virus testing, gene expression related to the immune response to COVID-19 and COVID-19 clinical manifestation.

Q3: How do the foci of genetic studies change during the COVID-19 crisis?

As indicated in Figure 5, the initial focus of COVID-19 genetic research is comparative studies between this novel coronavirus and previously discovered coronaviruses, including SARS-CoV and MERS-CoV. Starting from the mid-term of the COVID-19 crisis, this research focus diverges into the analysis of virus molecular structure and the human immune response. Further, derived from the direction of human immune response, vaccine development and gene expression related immunology become two emerging directions in this field.

Q4: Which specific genes are frequently highlighted and which ones are potentially emerging to COVID-19 genetic research?

In Figures 7, 8, we identified genes *ACE2, IL6, TMPRSS2*, and *TNF* as frequently highlighted ones in COVID-19 genetic research, and also identified genes, such as *FURIN, CXCL10, OAS1, OAS2, OAS3, ISG15*, etc., as emerging genes that may require further attention from the research community.

In terms of technical implications, our study provides a suite of intelligent bibliometric tools for biomedical researchers to conduct medical knowledge discovery. For example, it could profile the research landscape of a given medical case, with identified key players, research topics and highlighted associations between genes and diseases. Compared with other bibliometrics conducted on COVID-19, this work provides a systematic and adaptable research framework to profile the research landscape and exploit disease genetics-relate knowledge from literature. Additionally, this study specifically focused on COVID-19 genetic research and targeted a set of frequently highlighted genes and emerging genes on COVID-19, which could then turn to be the clue for COVID-19 prevention and treatment. The results of this study could benefit 1) clinical researchers with longitudinal analyses on COVID-19 genetic research, and 2) policymakers with insights in recognizing potential threats from the COVID-19 and providing pre-emptive actions on national strategies, science policy, and public health and administration for gene-level prevention and treatments.

There are also some limitations for future studies. From the methodological perspective, we designed a data-driven method to identify primary genes and emerging genes from the literature. However, integrating multiple data sources, such as clinical trials and curated medical knowledge databases, may gain value-added benefits. From the perspective of validation measurements, we employed evidence from the literature to interpret and support our findings with assistance from our medical experts. Nonetheless, the qualitative validation may be integrated with multiple quantitative measures, e.g., historical data-based validation, and expert knowledge-based scoring.

## Data Availability

The datasets presented in this study can be found at https://github.com/IntelligentBibliometrics/COVID-19-genetic-research.

## Footnotes

1 Detailed information on the PubMed database could be found on the website: https://pubmed.ncbi.nlm.nih.gov/

2 Input data and the results can be assessed through the link: https://github.com/IntelligentBibliometrics/COVID-19-genetic-research. Readers can use VOSViewer to reproduce these interactive maps and explore the detailed connections between a given node and others. The same for Figure 4.

3
https://www.ncbi.nlm.nih.gov/mesh/

4
https://ftp.ncbi.nih.gov/gene/DATA/GENE_INFO/Mammalia/Homo_sapiens.gene_info.gz

5
https://www.ncbi.nlm.nih.gov/snp/
